# Anterior Gastric Wall Excision and Retubularization: A Novel Procedure and Modification of Sleeve Gastrectomy for a Patient With Obesity

**DOI:** 10.7759/cureus.44057

**Published:** 2023-08-24

**Authors:** Oshiozimede Quincy Aigbonoga, Akarutu Andrew Okomayin, Ehichioya Charles Ikhifa

**Affiliations:** 1 Surgery/Plastic and Reconstructive Surgery, Irrua Specialist Teaching Hospital/Ambrose Alli University, Irrua, NGA; 2 Surgery/General Surgery, Irrua Specialist Teaching Hospital, Irrua, NGA; 3 Anaesthesiology, Irrua Specialist Teaching Hospital, Irrua, NGA

**Keywords:** modified sleeve gastrectomy, open bariatric surgery, weight loss, obesity surgery, obesity

## Abstract

Sleeve gastrectomy is a recognized surgical weight-loss procedure performed to reduce the amount of ingested food, thereby promoting a reduction in the patient’s weight. We present a 34-year-old multipara woman with complaints of abnormal excessive eating, progressive weight gain, and a body mass index (BMI) of 38.6 kg/m^2^. She was diagnosed with moderate obesity and received a modified sleeve gastrectomy using the partial anterior gastric excision and flap tubularisation technique. This case report presents the successful sleeving of the stomach using the anterior gastric wall excision with a gastric flap tubularisation technique.

## Introduction

According to the World Health Organization (WHO), obesity is characterized as a chronic illness pertaining to adults, adolescents, and children worldwide, defined by a measured body mass index (BMI) ≥ 30 kg/m^2^ [1]. It is estimated that 650 million adults are classified as obese worldwide, representing a staggering 13% of the adult population [2]. Despite obesity being a well-known ailment with a recognized impact on morbidity and mortality, the direction of treatment has largely stayed the same and is centered on weight loss [1]. While most people with obesity will achieve weight reduction through lifestyle and dietary modifications, a percentage of those classified as morbidly obese (BMI ≥ 40 kg/m^2^) will eventually require surgical intervention to minimize their mortality risk [1-3]. Currently, Roux-en-Y gastric bypass (RYGB), sleeve gastrectomy (SG), and adjustable gastric banding are the most popular and commonly performed bariatric surgeries (BS), and these procedures can be performed openly or as a laparoscopic procedure [2]. This case report discusses the outcome of a patient who received a modified open-sleeve gastrectomy with anterior gastric resection and gastric flap tubularization in our facility.

## Case presentation

The patient was a 34-year-old multipara lady presenting with an uncontrolled appetite of three years and excessive weight gain. Her uncontrolled appetite was characterized by an eating habit of six to eight meals per day versus her previous habit of two to three meals per day. About a year later, she noticed progressive weight gain, evidenced by comments from her relatives and the tightening of her previously well-fitted clothing. The patient also noticed progressively worsening feelings of heaviness, easy fatiguability on exertion, and reduced physical activity. She had received a laparotomy with a left ovarian cystectomy six years prior to presentation. She did not have diabetes mellitus or peptic ulcer disease, and she was neither hypertensive nor asthmatic. The patient has completed her family size, and her last menstrual period was six days before her presentation. She consumed alcohol occasionally.

She was young, afebrile, not pale, and was well-hydrated. Prior to surgery, her waist circumference was 117 cm, height 168 cm, and weight 109 kg, with a BMI of 38.6kg/m^2^. Her abdomen had multiple scarification marks, an inverted umbilicus, an extended midline infraumbilical scar that healed with secondary intention with the proximal end 12 cm from the xiphisternum and a sagging lower abdominal skin fold, with no areas of tenderness or palpable abdominal mass. An abdominopelvic ultrasound scan revealed essentially normal findings. Her laboratory workup showed a marginally elevated lymphocyte count and serum urea as well as marginally low levels of serum potassium and alkaline phosphatase (Table 1).

**Table 1:** Summary of the Results of the Laboratory Investigation

INVESTIGATION	RESULT	REFERENCE VALUE
Full Blood Count:		
Hemoglobin	12.8g/dL	11.0-15.0g/dL
Hematocrit	38.0%	33-45%
White Blood Cell Count	4,500/mm^3^	4,000-11,000/mm^3^
Neutrophil	44%	40-75%
Lymphocyte	55%	20-50%
Monocyte	1%	2-10%
Platelet Count	248,000/mm^3^	150,000-400,000/mm^3^
Serum Electrolyte:		
Sodium	138mmol/L	135-150mmol/L
Potassium	3.4mmol/L	3.5-5.5mmol/L
Chloride	102mmol/L	98-108mmol/L
Bicarbonate	26mmol/L	25-30mmol/L
Urea	56mmol/L	10-50mmol/L
Creatinine	83 µmol/L	45-110 µmol/L
Liver Function Test:		
Aspartate Transferase (AST)	10IU/L	0-47IU/L
Alanine Transferase (ALT)	7IU/L	0-49IU/L
Alkaline Phosphatase (ALP)	34IU/L	100-290IU/L
Total Bilirubin	13 µmol/L	3.5-17 µmol/L
Conjugated Bilirubin	2.5 µmol/L	0-5.0 µmol/L
Pregnancy Test:	Negative	
Fasting Blood Glucose:	103mg/dL	72-108mg/dL
Serum Protein:		
Total Protein	6,3g/dL	6.0-8.0g/dL
Albumin	4.4g/dL	3.0-5.0g/dL
Globulin	1.9g/dL	2.0-3.0g/dL

**Figure 1:**
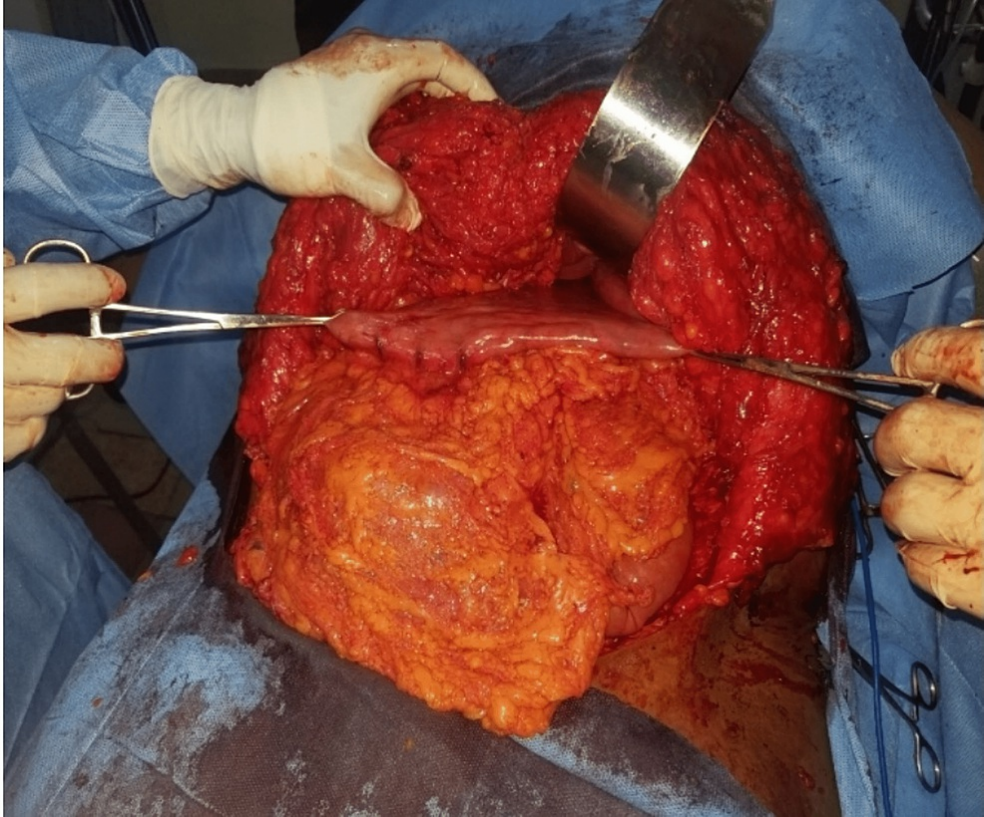
Intraoperative Gastric Mobilization

Prior to surgery, she consumed a liquid diet for 24 hours and fasted overnight. We also obtained informed consent from her. The surgery was performed under general anesthesia with cuffed endotracheal tube intubation, and anesthesia was maintained using propofol infusion. A size-18 nasogastric tube was passed. Antibiotic prophylaxis was achieved using intravenous meropenem (1 g) and intravenous metronidazole (500 mg). The skin was prepared using a 10% povidone-iodine solution, and appropriate sterile drapes were applied to expose the abdomen. The skin was marked before the incision was made, and then a lipodermal flap was raised. Recti muscles were separated via the midline raphe to access the peritoneal cavity. The falciform ligament was divided, both ends ligated using a Vicryl 1 suture, and the stomach was mobilized (Figure 1).

**Figure 2:**
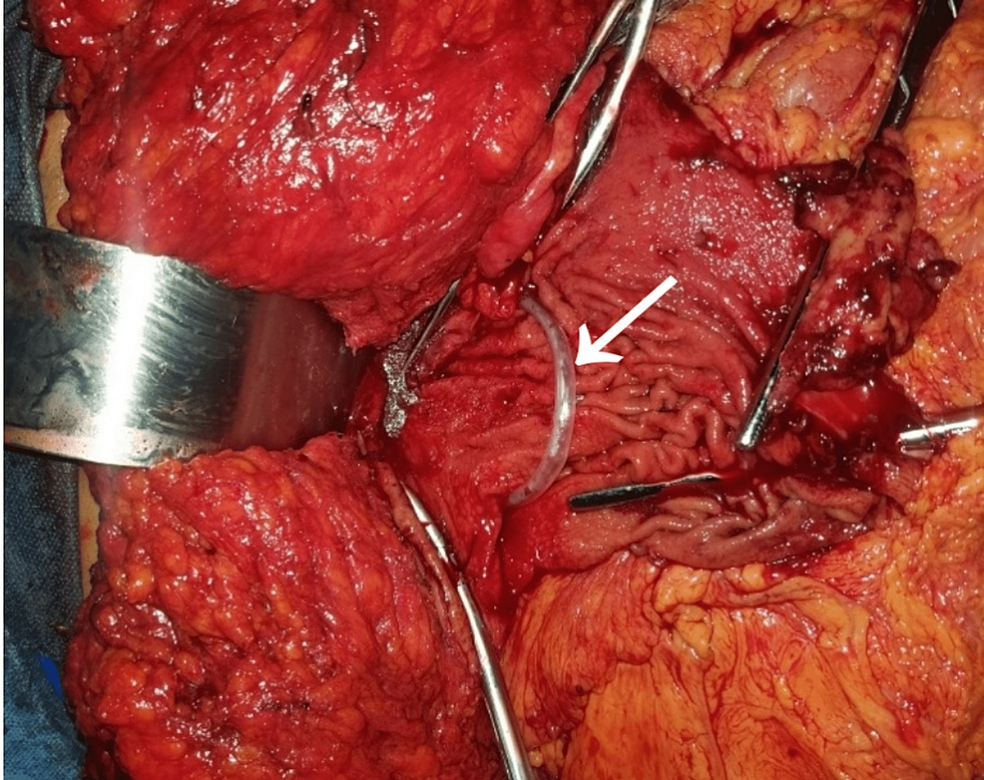
Residual Posterior Flap of the Stomach After Excision of the Anterior Wall and Before Retubularization Arrow pointing to size 16G nasogastric tube passing from the esophagus into the pylorus

The Crow’s feet, pylorus, and antrum were identified and mobilized. Thereafter, the greater omentum was reflected from the greater curvature of the stomach allowing adequate mobilisation and exposure. Following this, the short gastric vessels were identified and ligated using a Vicryl 2/0 suture. Hemostatic sutures were placed at points of proposed stab incisions. A non-crushing intestinal clamp was applied to the first part of the duodenum. Stab incisions were made on the anterior wall of the stomach. Non-crushing intestinal clamps were introduced into the stab incisions circumferentially to connect them, and then the encircled anterior aspect of the stomach was excised (Figure 2). Hemostasis was achieved by coagulation and ligature of bleeding vessels.

**Figure 3:**
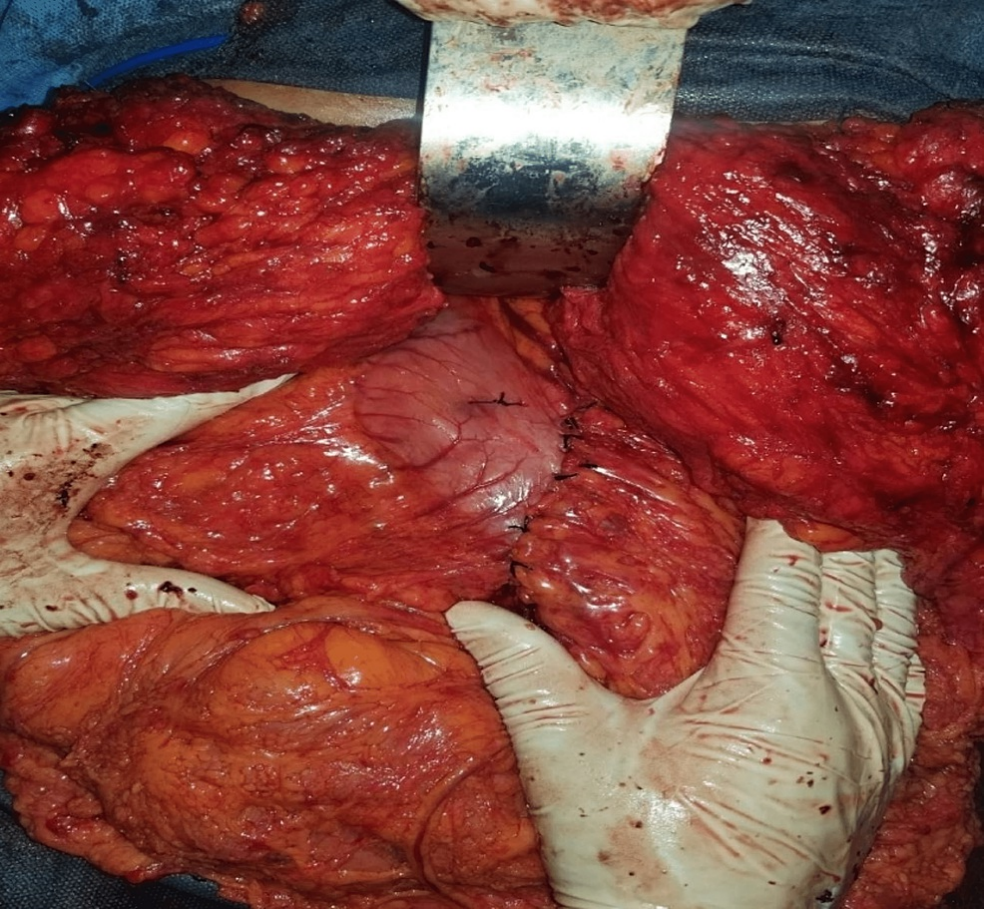
Intraoperative View of the Stomach After Excision of the Anterior Wall and Retubularization

The remnant stomach was retubularised in two layers using a Vicryl 0 suture, significantly reducing the stomach size (Figure 3). The repair was reinforced with topical surgical glue. A peritoneal drain was inserted, and the rectus fascia was closed using a Prolene 2 suture in a vertical mattress fashion. The abdominal wall was closed in layers using a Vicryl 0 suture in a simple interrupted fashion, and the skin closure was achieved with skin staples.

Postoperatively, the patient was placed on nil per os, IV fluids + IV vitamin C and B-complex, and continued on IV antibiotics (meropenem + metronidazole). She was started on IV omeprazole, IV analgesics (pentazocine, metamizole, and paracetamol, each alternated every four hours), IV Astymin, and deep vein thrombosis (DVT) prophylaxis. She had two pints of blood transfused because of a packed cell volume (PCV) of 26% on postoperative day (POD) 2; her post-transfusion PCV was 34%. The patient was started on fluid intake and liquid diets on POD 4. The peritoneal drain was discontinued on POD 5, and the subcutaneous drain was discontinued on POD 6. She was discharged home on POD 8 and started on a food intake schedule of a liquid diet for two weeks; a pureed diet (e.g., soup and milkshakes) for another four weeks; a soft diet (ekor, light amala, and semovita) for another four weeks; and a normal diet afterward.

**Figure 4:**
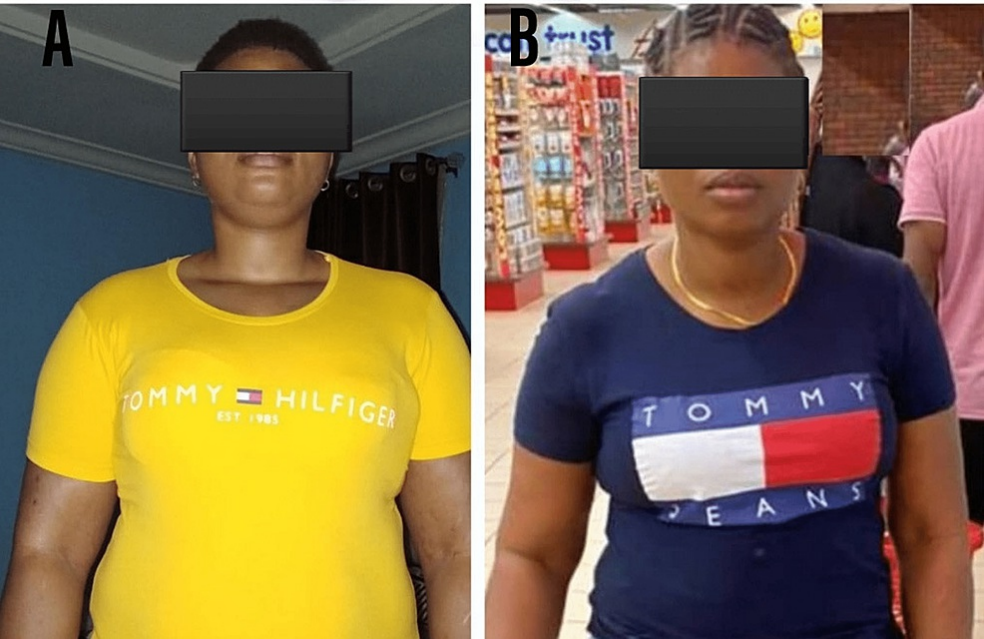
(A) Preoperative Appearance, (B): Sixth-Month Postoperative Appearance of the Patient

Her dietary intake dropped to an average range of 30 ml to 50 ml per meal. There was a progressive decrease in weight and BMI; 95 kg and 33.7 kg/m2, respectively, in the first-month postoperative period; 90 kg and 31.9 kg/m^2^ in the third-month postoperative period; and 80 kg and 28.3 kg/m^2^ in the sixth-month postoperative period (Figures 4A, 4B ). Waist circumference measured in the first- and sixth-month postoperative periods was 100 cm and 96 cm, respectively. She had no features suggestive of gastroesophageal reflux.

## Discussion

**Figure 5:**
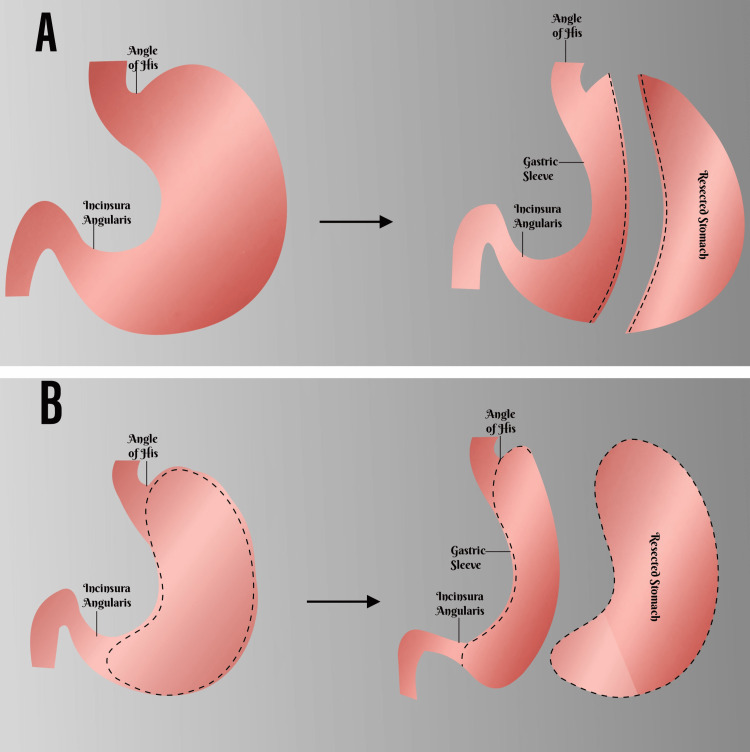
(A) Traditional Sleeve Gastrectomy, (B) Our Modification Original Creation. "Image Credits: Oshiozimede Quincy Aigbonoga"

Historically, sleeve gastrectomy was first performed as an open procedure in 1988 by Hess as part of his biliopancreatic diversion with a duodenal switch (BPD-DS) procedure [4]. With the evolution of laparoscopic surgery in the mid and late 1990s, Gagner performed the first laparoscopic sleeve gastrectomy as part of the BPD-DS in 1999 [4]. Over the next decades, sleeve gastrectomy was performed as the first of a two-stage bariatric procedure for super-obese patients (BMI > 60 kg/m^2^). However, in current practice, sleeve gastrectomy has evolved to be a standalone bariatric procedure owing to its many advantages [5]. Traditionally, sleeve gastrectomy is essentially a primarily restrictive bariatric procedure consisting of a left partial gastrectomy of the fundus and body and tubularisation of the remnant gastric tissue along the lesser curvature (Figure 5A) [5,6]. In our case, we decided to proceed with a modified open-sleeve gastrectomy that involved anterior gastrectomy with tubularization of the posterior gastric flap (Figure 5B).

The indication for metabolic and bariatric surgery is individuals with a BMI of 35 kg/m^2^ or higher regardless of the presence, absence, or severity of obesity-related conditions [5]. It should also be considered for people with a BMI of 30-34.9 kg/m^2^ and metabolic disease and in ‘appropriately selected children and adolescents’ [7]. The comorbidities include hypertension, diabetes mellitus, and polycystic ovarian syndrome [7-9]. Other indications include interference with daily activities and patient requests.

This report describes the case of a 34-year-old woman with complaints of abnormal excessive eating habits, interference with normal daily activities, and a BMI of 38.6 kg/m^2^. These complaints necessitated her presentation for gastric volume reduction surgery. In this case, we decided to proceed with a modified open-sleeve gastrectomy that involved anterior gastrectomy with tubularization of the posterior gastric flap (Figure 5B). This modification permitted the placement of the suture line along the lesser curvature away from the most dependent part of the new stomach, thereby reducing the chance of postoperative leak [9,10]. The two-layer wound closure technique and surface reinforcement with surgical glue are believed to further prevent postoperative bleeding and leaks [4].

The surgical principles employed in the planning and implementation of this new surgical technique of gastric sleeving were in line with the recommendations of the International Sleeve Gastrectomy Expert Panel Consensus Statement [4]. These best-practice guidelines were drawn out from the experience of 12,000 cases performed in 24 bariatric surgery centers across 11 countries. In line with this consensus statement, the Crow’s feet, pylorus, and gastric antrum were appropriately identified, following which the greater omentum was reflected from the greater curvature of the stomach and the short gastric vessels were ligated. These steps ensured adequate mobilization of the stomach and control of primary hemorrhage during gastric resection. Also, the stomach closure was performed in a two-layer fashion and further reinforced with surgical glue. This is in line with the staple line reinforcement to prevent bleeding and leaks. This modification allows the siting of the suture line along the lesser curvature and away from the most dependent part of the new stomach. This is the main feature of this new technique and is thought to avoid the slightest possibility of a leak, which is a feared complication of sleeve gastrectomy [10].

## Conclusions

To our knowledge, this is the first description of a modified open-sleeve gastrectomy using the anterior gastric wall excision and tubularization of the residual gastric flap with remarkable short-term results in our environment. In the context of recently available literature, our opinion is that this novel procedure is an easy, optimal, and safe weight loss surgical procedure with theoretically comparable outcomes in patients with obesity requiring weight loss surgery. The surgical technique employed the principles of standardization proposed by the expert committee recommendations of 2012 in Florida. However, a larger patient population and longer follow-up will be required to propose it as a first-line, stand-alone bariatric procedure.
